# Estimates of healthcare utilisation and deaths from waterborne pathogen exposure in Ontario, Canada

**DOI:** 10.1017/S0950268820000631

**Published:** 2020-03-13

**Authors:** Susan Lavinia Greco, Christopher Drudge, Reisha Fernandes, JinHee Kim, Ray Copes

**Affiliations:** 1 Environmental and Occupational Health, Public Health Ontario, Toronto, Ontario, Canada; 2 Dalla Lana School of Public Health, University of Toronto, Toronto, Ontario, Canada; 3 School of Population and Public Health, University of British Columbia, Vancouver, British Columbia, Canada

**Keywords:** burden of disease, deaths, gastrointestinal illness, hospitalisations, opportunistic premise plumbing pathogens, waterborne pathogens

## Abstract

Burden of disease analyses can quantify the relative impact of different exposures on population health outcomes. Gastroenteritis where the causative pathogen was not determined and respiratory illness resulting from exposure to opportunistic pathogens transmitted by water aerosols have not always been considered in waterborne burden of disease estimates. We estimated the disease burden attributable to nine enteric pathogens, unspecified pathogens leading to gastroenteritis, and three opportunistic pathogens leading primarily to respiratory illness, in Ontario, Canada (population ~14 million). Employing a burden of disease framework, we attributed a fraction of annual (year 2016) emergency department (ED) visits, hospitalisations and deaths to waterborne transmission. Attributable fractions were developed from the literature and clinical input, and unattributed disease counts were obtained using administrative data. Our Monte Carlo simulation reflected uncertainty in the inputs. The estimated mean annual attributable rates for waterborne diseases were (per 100 000 population): 69 ED visits, 12 hospitalisations and 0.52 deaths. The corresponding 5th–95th percentile estimates were (per 100 000 population): 13–158 ED visits, 5–22 hospitalisations and 0.29–0.83 deaths. The burden of disease due to unspecified pathogens dominated these rates: 99% for ED visits, 63% for hospitalisations and 40% for deaths. However, when a causative pathogen was specified, the majority of hospitalisations (83%) and deaths (97%) resulted from exposure to the opportunistic pathogens *Legionella* spp., non-tuberculous mycobacteria and *Pseudomonas* spp. The waterborne disease burden in Ontario indicates the importance of gastroenteritis not traced back to a particular pathogen and of opportunistic pathogens transmitted primarily through contact with water aerosols.

## Introduction

Gastrointestinal (GI) illness from exposure to pathogens in water can result from factors such as weather events, the failure of water treatment or distribution systems, and human error [1]. Acute GI illness has been associated with pathogenic contamination of drinking water according to self-reported information from telephone surveys [2], though there is less information on illnesses requiring medical care. Similarly, there tends to be more information on outbreaks (e.g. [3, 4]) than on endemic rates. In Canada, previous estimates of the health burden of waterborne disease and surveillance efforts (e.g. [5–9]) have focused on enteric pathogens introduced via faecal contamination, such as *Giardia* spp., *Cryptosporidium* spp. and *Escherichia coli*. Exposure to these pathogens occurs via ingestion. However, there is growing concern about pathogens that are naturally occurring in water and can be transmitted by inhalation of aerosols [10–14]. Such pathogens include *Legionella* spp. and *Pseudomonas* spp., which are naturally occurring in water and not correlated with faecal indicators often used to assess water quality [15]. These two pathogens along with non-tuberculous mycobacteria (NTM) were collectively associated with 91% of deaths from diseases transmitted by water in the USA [16].

Pulmonary infections are the principal outcome of NTM exposure, with a much smaller number of extrapulmonary infections (e.g. skin) resulting from exposure to this pathogen [12]. Pontiac fever (mild flu-like illness) or the more serious Legionnaires' disease can result from *Legionella* spp. exposure. Exposure to *Pseudomonas* spp. can result in pneumonia, as well as blood and skin infections. *Pseudomonas* spp. can also be transmitted by direct contact with water and healthcare workers [11]. These three pathogens are able to resist disinfection, grow in biofilm and amoebae and survive in stagnant (low oxygen) water [17]. They can reside in drinking-water distribution systems and premise plumbing (the portion of the water distribution system inside buildings) [11]. The three pathogens have been referred to as opportunistic premise plumbing pathogens because of the attributes listed above and their tendency to infect individuals who are older, immunosuppressed and/or have pre-existing chronic diseases [18].

The number of notifiable cases of illness reported to public health authorities and captured in surveillance systems is typically fewer than the number of people who become ill, seek medical care and receive confirmatory laboratory diagnostic testing [19]. Even when diseases of public health significance are reported, the exposure source (e.g. food, animal, water) is often unclear or unknown [9], making it difficult to attribute the reported cases to any particular source. In addition, surveillance may be based on the identification of specific pathogens, even though many cases may never be traced back to a specific pathogen. Previous studies found the burden of foodborne illness not traced back to a particular pathogen was 1.3–4.1 times larger than the total foodborne burden attributed to major known pathogens [20, 21].

Researchers have estimated emergency department (ED) visits and hospitalisations in the year 2013 [22] and deaths from 2003 to 2009 [16] for select diseases that can be transmitted by water in the USA. The studies, which made use of surveillance, administrative and death certificate data for disease estimates, found most of the disease was not due to enteric pathogens. Both studies noted their estimates did not account for the portion of disease transmitted by water as opposed to other sources (e.g. food, human, animal) and suggested that an attribution process was a necessary next step for a comprehensive analysis.

Burden of disease analyses, which involve attributing a fraction of disease outcomes (e.g. deaths) to an exposure or source, can be useful for understanding the relative impact of different environmental exposures on population health [23, 24]. We estimated the burden of disease from exposure to waterborne pathogens in Ontario, Canada (population ~14 million) as part of a larger effort to understand the relative burdens from significant environmental exposures. We were interested in the portion of disease outcomes attributable to water for (1) specific enteric pathogens, (2) unspecified pathogens leading to GI illness and (3) pathogens largely transmitted through inhalation of water aerosols. Taking advantage of comprehensive administrative health databases, we evaluated the burden in terms of ED visits, hospitalisations and deaths; measures reflecting moderate-to-severe outcomes and readily understood by decision-makers.

## Methods

We followed a framework similar to other environmental burden of disease studies (e.g. [23, 24]). After selecting the pathogens of interest, we used diagnostic codes to quantify the diseases of interest in terms of counts and crude rates. Then, we attributed a fraction of the disease to the waterborne transmission route. We used a probabilistic model and inputs as described below.

### Selection of waterborne pathogens

To select the waterborne pathogens to include in our analysis, we first examined surveillance-derived annual case rates (2006–2015) representing laboratory-identified cases of reportable diseases in Ontario [25]. We next reviewed modelled estimates of annual hospitalisations and deaths, for 19 bacteria, five parasites and six viruses in Canada (2000–2010) [21]. After ranking the estimates based on the expected contribution from waterborne transmission, we selected 10 pathogens that were among the top of either list, including nine enteric pathogens and *Legionella* spp.

**Fig. 1.**
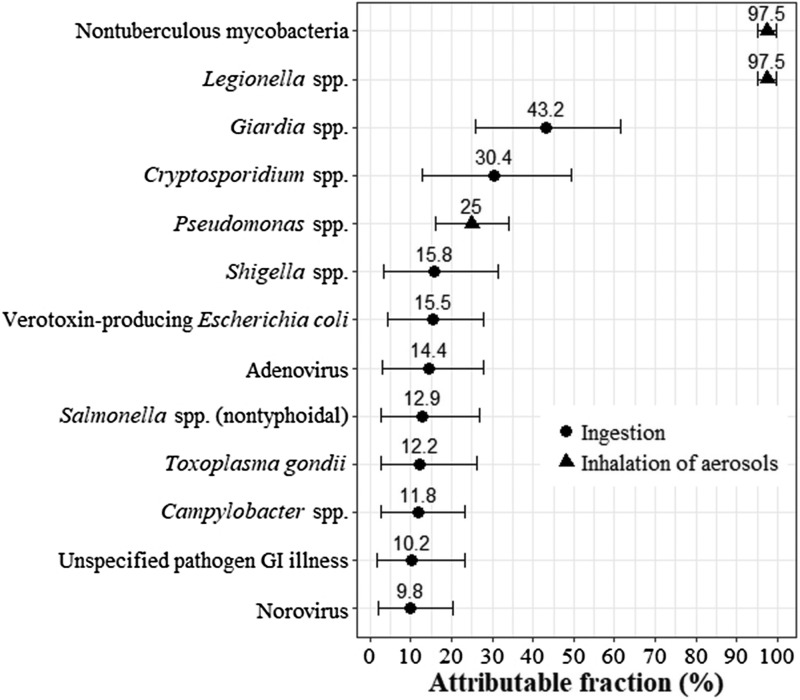
Fraction of disease attributable to water transmission by a pathogen with thepredominant route of exposure (ingestion or inhalation) indicated. Mean shown by dot (for pathogens that are ingested) or triangle (for pathogens that are inhaled) and 5th and 95th percentiles shown by whiskers. GI, gastrointestinal.

We next reviewed the indexed scientific literature and consulted with clinicians to augment our pathogen list. We added *Pseudomonas* spp. and NTM based on their expected contributions to hospitalisations and deaths in the province [12, 26]. We also added a general category for gastroenteritis not traced back to a specific pathogen (hereafter called ‘unspecified pathogen GI illness’) since causative pathogens are not identified in all cases. Illustrating this, in the USA, 80% of foodborne illness was due to unspecified agents, including known agents with insufficient data for estimating agent-specific illness, and 20% was due to major known pathogens [20]. Figure 1 contains a list of the 12 pathogens (plus unspecified pathogen GI illness) we included in our study.

### Calculation of waterborne disease burden

The waterborne attributable disease burden (AD) is the waterborne attributable fraction (AF) multiplied by the disease count (D): AD = AF × D. The waterborne attributable disease (crude) rate burden (ADR) is the attributable disease divided by the population (P): ADR = AD/P. The Ontario population for the year 2016 was 13 875 394 [27].

We conducted a Monte Carlo analysis (10 000 iterations) using @RISK, a Microsoft Excel add-in (version 7.5, Palisade Corporation, USA). Attributable burden counts and rates were summarised using the simulation mean and 5th and 95th percentile estimates. The number of iterations was sufficient for convergence of the results. We also fit distributions to attributable fraction inputs using @RISK and selecting the fit with the lowest AIC.

### Attributable fractions

All attributable fraction distributions are summarised in Figure 1 and fully specified in Table A1. We relied on a Canadian expert elicitation study to develop the attributable fractions for the nine enteric pathogens included in this study. One study compiled estimates from 31 experts on the major transmission routes (foodborne, waterborne, animal contact, person-to-person and other) for 28 enteric pathogens [28]. They combined expert estimates using triangular probability distributions. Rather than using their pathogen-specific results for the waterborne transmission route directly, we constrained each waterborne attributable fraction to be consistent with the attribution from the other four major transmission routes (i.e. all routes summed to 100%) and used the best fitting distribution type for each pathogen.

We developed the remaining four attributable fractions from other literature sources and clinical input. Water is the major environmental reservoir and water aerosols the main source of exposure for *Legionella* spp. and NTM in Canada [29], although cases of the disease can also occur via aerosolised soil [30, 31]. As such, we attributed 95–100% of infections to waterborne transmission for these pathogens. *Pseudomonas* spp. infections are often healthcare-acquired, with transmission routes including the hands of healthcare workers, direct patient-to-patient contact and contamination of the environment (e.g. faucets, sink drains) and equipment [32, 33]. We attributed 15–35% of *Pseudomonas* spp. infections to waterborne transmission based on our review of the literature (e.g. systematic review of its transmission in hospital intensive care units [34]) and input from Ontario-based infectious disease experts.

For unspecified pathogen GI illness, we developed an attributable fraction by fitting a distribution to the mean estimates of the proportion of GI illness attributable to waterborne pathogens in Canada that were published in the last 10 years [8, 9, 35–37]. The mean estimates, ranging from 2% to 20%, were based either on selected groups of known pathogens for which surveillance data were available or the risk of acute GI illness attributable to drinking water.

### Disease counts

We expressed disease in terms of deaths and healthcare utilisation as their importance is readily understood and reliable data exist for nearly all Ontario residents. We obtained counts of deaths, hospitalisations and ED visits corresponding to specific disease diagnoses. For unspecified pathogen GI illness, we were also able to estimate the counts of physician office visits using the physician billing claims database for the province (Ontario Health Insurance Plan; see [38] for method). However, we do not present these results formally because there was insufficient detail to provide estimates at the pathogen level.

The diagnostic codes shown in Table 1 are from the *International Statistical Classification of Diseases, 10th Revision* (ICD-10) or ICD-10-CA (the Canadian modification of ICD-10). Most diseases were captured by one or two diagnosis codes. We also included Guillain–Barré syndrome due to *Campylobacter* spp. and haemolytic–uraemic syndrome due to verotoxin-producing *E. coli* (VTEC) because of their expected major contribution to the burden for these pathogens. The disease counts reflect the year 2016 (the latest year for which full data were available). Additionally, given the sporadic nature of outbreaks, we examined disease counts over the most recent 5-year period (2012–2016), though we did not identify any discernible trends over that period (Tables A2a–c).

**Table 1.** Emergency department visit, hospitalisation and death counts corresponding to diagnosis codes (from all exposures) in Ontario for the year 2016

Disease	Diagnosis code(s)	ED visits^a^^,^^b^	Hospitalisations^a^^,^^c^	Deaths^a^^,^^d^
		Min/Max	Min/Max	Min/Max
Adenovirus intestinal infection	A08.2	–/–	31/57	–/–
*Campylobacter* spp. intestinal infection (campylobacteriosis)	A04.5	169/184	140/176	–/–
*Campylobacter-*associated GBS^e^	G61.0^f^	23/31	64/91	–/7
*Cryptosporidium* spp. intestinal infection (cryptosporidiosis)	A07.2	19/23	–/11	–/–
*Giardia* spp. intestinal infection (giardiasis)	A07.1	30/35	11/25	–/–
*Legionella* spp. infection (legionellosis)	A48.1, A48.2	–/7	81/120	–/8
NTM infection^e^	A31.x	24/55	78/455	13/49
Norovirus intestinal infection	A08.1	32/37	44/109	–/–
*Pseudomonas* spp. pneumonia and sepsis^e^	A41.51^g^, J15.1	31/45	423/1293	7/47^g^
*Salmonella* spp. (non-typhoidal) infection (salmonellosis)	A02.x	208/252	290/386	–/–
*Shigella* spp. intestinal infection (shigellosis)	A03.x	31/34	24/36	–/–
*Toxoplasma gondii* infection (toxoplasmosis)	B58.x	7/9	–/27	–/–
VTEC intestinal infection	A04.3	–/–	13/16	–/–
VTEC-associated HUS^e^	D59.3^f^	6/7	24/59	–/–
Unspecified pathogen GI illness^e^	A04.9, A07.9, A08.4, A09, A09.0, A09.9^h^, K52.9^h^, R19.x^h^	88 358/103 074	7106/16 525	165/399

GBS, Guillain–Barré syndrome; HUS, haemolytic–uraemic syndrome; Max, maximum; Min, minimum; NTM, non-tuberculous mycobacteria; VTEC, verotoxin-producing *Escherichia coli*.

a Counts with small cell sizes (<6) were suppressed; indicated by a dash.

b Minimum was for the main problem only. Maximum was for any diagnosis type. ICD-10-CA diagnosis code used.

c Minimum was for the most responsible diagnosis only. Maximum was for any diagnosis type. ICD-10-CA diagnosis code used.

d Minimum was for the underlying cause of death only. Maximum was for any cause of death. ICD-10 diagnosis code used.

e Selected based on literature survey and clinical expert judgment; all others selected by the examination of surveillance case and modelled hospitalisation/death data (see methods for details).

f The diagnosis code captured all causes of the syndrome, so we estimated the proportion due to the specified pathogen for each year. The proportions were 0.09–0.47 (uniform distribution) for *Campylobacter*-associated GBS and 0.75–1.00 (uniform distribution) for VTEC-associated HUS (see Drudge *et al*. [38] for details).

g The diagnosis code A41.51 (sepsis due to *Pseudomonas*) was not included in ICD-10 (used to describe deaths), so we estimated the proportion of deaths captured by ICD-10 code A41.5 (sepsis due to other Gram-negative organisms) that were specifically due to *Pseudomonas* spp. sepsis. We used the proportion of hospitalisations captured by ICD-10-CA code category A41.5 ×  that specifically were A41.51 (sepsis due to *Pseudomonas*) (most responsible diagnosis only).

h These diagnosis codes collectively captured gastroenteritis from any cause, so we estimated the proportion not due to pre-existing conditions (e.g. irritable bowel syndrome). The proportion was 0.81–0.88 (uniform distribution) (see Drudge *et al*. [38] for details).

*Note*: in 2016, there were 449 656 physician office visits billed for gastroenteritis (Ontario Health Insurance Plan diagnostic code 009). After applying footnote (h), the central estimate is 379 959 visits.

For all Ontario residents, we acquired annual counts (de-identified aggregate data) from the National Ambulatory Care Reporting System, the Discharge Abstract Database and the Ontario vital statistics death data. Since any health record may include multiple diagnoses, we modelled the disease counts as uniform distributions as follows. For deaths, the minimum count reflects the underlying cause of death only, while the maximum reflects any cause of death (i.e. underlying, immediate, all antecedent and other significant contributing). For hospitalisations or ED visits, the minimum count reflects the ‘most responsible diagnosis’ or the ‘main problem’, respectively, while the maximum reflects any diagnosis type. All disease count characterisations are provided in Table 1.

## Results

The estimated attributable disease rates for deaths and health care utilisation are presented in Table 2. We presented estimates where the mean attributable rate (per 100 000 population) was at least 0.01 for deaths, 0.1 for hospitalisations or 0.1 for ED visits. These rates roughly correspond to one death and 10 hospitalisations or ED visits in the study population and are referred to as the presentation thresholds. Seven of the 13 pathogen groups met at least one of the presentation thresholds: unspecified pathogen GI illness, all three pathogens transmitted by inhalation of aerosols and three of the nine enteric pathogens. We also include the rates for the sum of the six enteric pathogens that did not meet any presentation threshold. Overall, the mean attributable rates were 69 (5th and 95th percentiles: 13, 158) per 100 000 for ED visits, 12 (5th and 95th percentiles: 5, 22) per 100 000 for hospitalisations and 0.52 (5th and 95th percentiles: 0.29, 0.83) per 100 000 for deaths attributable to waterborne pathogens in Ontario. In Ontario, the mean attributable estimates corresponded to 9600 ED visits, 1600 hospitalisations and 70 deaths for the year 2016 (Table A3). The ratio of ED visits to hospitalisations to deaths was approximately 130:20:1.

**Table 2.** Estimated attributable ED visit, hospitalisation and death rates (per 100 000 population) to identify waterborne pathogens and unspecified waterborne pathogens causing GI illness in Ontario in the year 2016

Disease	Attributable ED visit rate^a^	Attributable hospitalisation rate^a^	Attributable death rate^a^
	Mean (5th, 95th)^b^	Mean (5th, 95th)^b^	Mean (5th, 95th)^b^
Unspecified pathogen GI illness	68 (12, 157)	7.5 (1.3, 17.8)	0.21 (0.03, 0.50)
NTM infection	0.24 (0.17, 0.34)	1.4 (0.6, 2.6)	0.22 (0.10, 0.33)
*Pseudomonas* spp. pneumonia and sepsis	<0.1	1.3 (0.6, 2.2)	0.05 (0.01, 0.09)
*Legionella* spp. infection	<0.1	0.66 (0.58, 0.78)	0.04 (0.02, 0.05)
*Salmonella* spp. (non-typhoidal) infection	0.21 (0.04, 0.43)	0.30 (0.06, 0.62)	<0.01
*Campylobacter* spp. intestinal infection and associated GBS	0.17 (0.04, 0.34)	0.19 (0.04, 0.39)	<0.01
*Giardia* spp. intestinal infection	0.10 (0.06, 0.14)	<0.1	<0.01
Sum for pathogens below presentation threshold^c^	0.13 (0.08, 0.18)	0.20 (0.12, 0.29)	0.01 (0.00, 0.01)
Sub-total all enteric pathogens^d^	*0.60 (0.37, 0.88)*	*0.74 (0.42, 1.12)*	*0.01 (0.01, 0.02)*
Sub-total for inhaled aerosol pathogens^e^	*0.34 (0.26, 0.44)*	*3.4 (2.2, 4.8)*	*0.31 (0.18, 0.43)*
Sub-total all identified pathogens^f^	*0.94 (0.69, 1.23)*	*4.1 (2.9, 5.6)*	*0.32 (0.20, 0.44)*
Total	69 (13, 158)	12 (5, 22)	0.52 (0.29, 0.83)

ED, emergency department; GBS, Guillain–Barré syndrome; GI, gastrointestinal; NTM, non-tuberculous mycobacteria.

a Results presented when the rate per 100 000 population was at least 0.1 ED visits, 0.1 hospitalisations or 0.01 deaths. The sum of the pathogens that did not meet the presentation threshold is shown as a separate row. Results may not add up due to rounding (to two significant figures).

b The simulation is summarised by the mean and 5th and 95th percentiles of the 10 000 iterations.

c This is the sum for the six pathogens that were below the presentation threshold (and thus not included elsewhere in this table): adenovirus, *Cryptosporidium* spp., norovirus, *Shigella* spp., *Toxoplasma gondii* and verotoxin-producing *Escherichia coli.*

d This is a sum of all enteric pathogens: adenovirus, Campylobacter spp., Cryptosporidium spp., Giardia spp., norovirus, Salmonella spp., Shigella spp., *Toxoplasma gondii* and verotoxin-producing *Escherichia coli*.

e This is the sum for the largely inhaled pathogens *Legionella* spp. infection, NTM infection and *Pseudomonas* spp. pneumonia and sepsis from the rows above.

f This is the sum for all identified pathogens. It includes all nine enteric pathogens (including those below the presentation threshold) and all three opportunistic (respiratory) pathogens, but excludes unspecified pathogen GI illness.

*Note*: we estimated a physician office visit rate per 100 000 for unspecified pathogen GI illness of 280 (5th and 95th percentiles: 50 630).

The estimated attributable rates for GI illness without a specified pathogen were strikingly higher than for any identified pathogen, and in some cases, higher than the sum of all 12 identified pathogens. At the mean, the attributable rate of unspecified pathogen GI illness was over 70 times higher for ED visits compared to the sum of all 12 identified pathogens (68 compared to 0.94 per 100 000). For hospitalisations, the mean was nearly twice as high (7.5 *vs.* 4.1 per 100 000), while for deaths, the mean rate was lower (0.21 *vs.* 0.32 per 100 000).

When considering only identified pathogens, the results varied by the type of healthcare encounter. The attributable rates for hospitalisations and deaths were highest for NTM, *Pseudomonas* spp. and *Legionella* spp. The sum of these three pathogens represented 83% of hospitalisations and 97% of deaths for identified pathogens. Furthermore, the pathogen NTM had the highest attributable ED visit rate. For ED visits, all enteric pathogens comprised 65% of the sub-total for identified pathogens. While there was some year-to-year variability, the rankings of the top pathogens, based on the mean attributable rates, did not change over the years 2012–2016 (underlying data in Tables A1 and A2a–c).

**Fig. 2.**
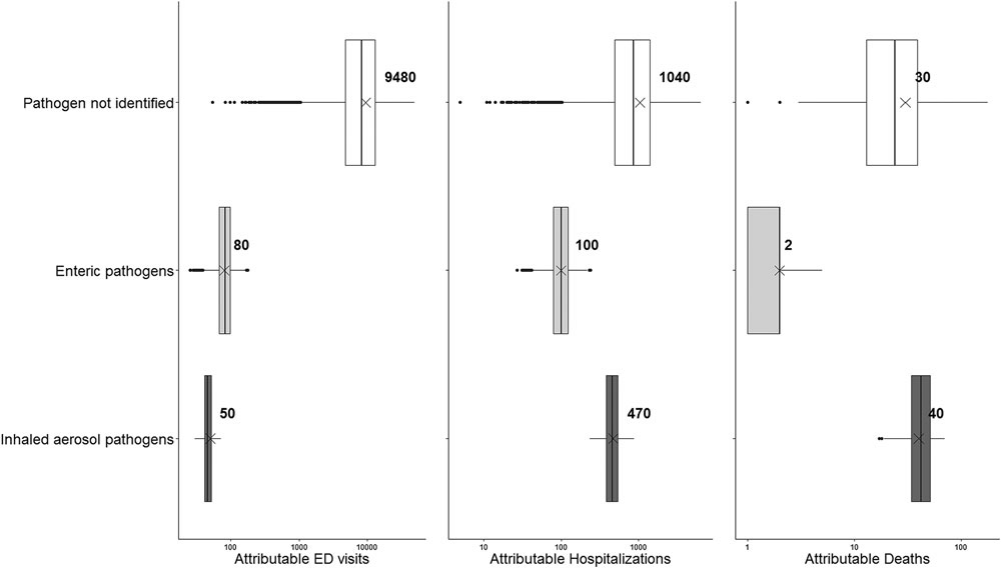
Estimates of emergency department (ED) visits, hospitalisations and deaths attributable to gastroenteritis when the causative pathogen was (a) not identified, (b) enteric pathogens or (c) inhaled aerosol pathogens. *Notes*: ‘Pathogen not identified’ reflects the estimates of healthcare utilisation and deaths where the causative pathogen was not identified. ‘Enteric pathogens’ represent the sum of adenovirus, *Campylobacter* spp., *Cryptosporidium* spp., *Giardia* spp., norovirus, *Salmonella* spp., *Shigella* spp., *Toxoplasma gondii* and verotoxin-producing *Escherichia coli*. ‘Inhaled aerosol pathogens’ represent the sum of *Legionella* spp., non-tuberculous mycobacteria and *Pseudomonas* spp. The box encloses the 25th to 75th percentile simulation results for attributed ED visits, hospitalisations or deaths. The line in each box represents the median of the distribution, while the ‘*x*’ represents the mean. The whiskers represent 1.5 times the interquartile range and points beyond this range indicate outliers.

Figure 2 shows how the results vary by the type of healthcare encounter when the results are split into three categories: unspecified pathogens, enteric pathogens and inhaled aerosol pathogens. Again, the mean burden attributable to unspecified pathogens is greatest for ED visits and hospitalisations, while the mean attributable burden to inhaled aerosol pathogens is greatest for deaths. The mean attributable burden for enteric pathogens was lowest for all types of healthcare encounter.

## Discussion

This work estimated the burden of disease for 12 pathogens and for unspecified pathogen GI illness transmitted through water in Ontario, Canada. It provides several unique contributions to the literature. First, it uses comprehensive administrative data to quantify healthcare utilisation and mortality, and apportions a fraction of these outcomes to the waterborne route of transmission. Second, it highlights the importance of waterborne GI illness where no causative pathogen was identified as well as infections transmitted through inhalation of water aerosols. Estimates of pathogen-specific enteric illness do not include these important contributors to waterborne disease. Third, it provides estimates of annual ED visits, hospitalisations and deaths attributable to infections transmitted through water that can be compared with other environmental hazards and over time. This analysis is part of a larger initiative by Public Health Ontario to estimate the environmental burden of disease in the province in order to compare the relative mortality and healthcare burden across a suite of environmental hazards using a consistent framework. The results can inform local and provincial decision-making regarding environmental, public health and planning priorities, with the ultimate goal of protecting and improving the health of the population.

We did not report individual results for the burden attributable to adenovirus, *Cryptosporidium spp.*, norovirus, *Shigella spp.*, *Toxoplasma gondii* or VTEC as the estimates did not meet our reporting threshold. We did not locate other burden estimates for these pathogens, save a study [39] that assessed the burden of *Cryptosporidium* spp. mortality in China by quantitative microbial risk assessment (QMRA) and also estimated a low attributable death rate (range: 0.011–0.17 per 100 000 depending on the treatment type used). Differences in the estimation approach can partially explain why these estimates are higher than our corresponding values (they did not meet the reporting threshold for the mortality of 0.01 per 100 000).

We compared our reported results with others available for Canada and the USA to provide context. The corresponding crude hospitalisation and death rates are shown in Table 3. The Global Burden of Disease 2015 Study reported an unsafe water, sanitation and handwashing mean death rate for Canada of 0.56 per 100 000 [6], which is about 8% higher than our mean waterborne attributable death rate. This is a relatively close level of agreement, considering differences in inputs, exposures and diseases considered, and models. As expected, our estimates of healthcare utilisation and deaths are lower than other reported estimates for the total number of cases of waterborne infections. Supporting this finding, telephone surveys to determine the levels of acute GI illness in Canada found that only 9–22% of respondents who reported illness sought medical care [40, 41]. One study estimated acute GI illness in Canada by modelling data on drinking-water treatment systems, randomised control trials of drinking-water interventions and GI illness risk associated with distribution systems [8]. They estimated approximately 1200 cases per 100 000 population, which is over four times greater than our rate of 280 physician office visits per 100 000 population for unspecified pathogen GI illness (see note under Table 2). Since our approach only captures cases who had contact with the healthcare system, our results should be considered an underestimate of the total number of illnesses from waterborne pathogens.

**Table 3.** Comparison of results from this study (year 2016) to comparable crude hospitalisation and death rates from other studies and surveillance data

Exposure	Hospitalisation rate per 100 000	Death rate per 100 000	Source
Waterborne illness	NE	0.56	GBD 2015 estimate for unsafe water, sanitation and handwashing for Canada [5]
Foodborne illness	50^a^	0.51^a^	Drudge *et al*. [38] estimate for foodborne illness in Ontario
Waterborne illness	39^b^	2.3^b^	Table 6 of Adam *et al*. [22] for hospitalisations and Table 5 of Gargano *et al*. [16] for deaths, converted to crude rates
**Waterborne illness**	**12**	**0.52**	**Present study**
Unspecified GI illness	13^c^	0.23^c^	Thomas *et al*. [21] unspecified estimate for all transmission routes, multiplied by our waterborne transmission attributable fraction
Unspecified GI illness	9.5^c^	0.13^c^	Scallan *et al*. [20] unspecified estimate for all transmission routes, multiplied by our waterborne transmission attributable fraction
**Unspecified GI illness**	**7.51**	**0.21**	**Present study**
*Pseudomonas* spp.	17^b^	1.6^b^	Table 1 of Adam *et al*. [22] for hospitalisations and Table 5 of Gargano *et al*. [16] for deaths, converted to crude rates
***Pseudomonas* spp.**	**1.3**	**0.05**	**Present study**
NTM	4.0^b^	0.41^b^	Table 1 of Adam *et al*. [22] for hospitalisations and Table 5 of Gargano *et al*. [16] for deaths, converted to crude rates
**NTM**	**1.4**	**0.22**	**Present study**
*Legionella* spp.	0.82^d^	0.07^d^	OAHPP (2019) reflecting 2016 provincial surveillance data
*Legionella* spp.	0.97^b^	0.08^b^	Table 1 of Adam *et al*. [22] for hospitalisations and Table 5 of Gargano *et al*. [16] for deaths, converted to crude rates
***Legionella* spp.**	**0.66**	**0.04**	**Present study**

GI, gastrointestinal; NTM, non-tuberculous mycobacteria; NE, not estimated.

a This estimate for foodborne illness can be used to compare the foodborne and waterborne transmission routes for Ontario.

b These estimates are *not* specific to waterborne transmission. The crude rates were calculated using a population estimate for the USA of 316.2 million (year 2013) for Adam *et al*. [22] and of 298.4 million (year 2006) for Gargano *et al*. [16]. Table 1 of Adam *et al*. [22] reported a hospitalisation total for 13 pathogens that converts to 32 per million and is used as the total when examining the contribution of specific pathogens.

c We obtained or back-calculated the unspecified estimates for all transmission routes and reflecting both domestic and travel-acquired illness. We then multiplied the all transmission route estimate from the studies by our unspecified GI illness attributable fraction (mean of 0.102) for waterborne transmission.

d These estimates are *not* specific to waterborne transmission. They reflect confirmed and probable cases reported to public health authorities in 2016. Hospitalisations reflect admissions up to 60 days before or 90 days after the episode. Deaths reflect any cause of death, unless there was an indication that the reportable disease was unrelated to the cause of death.

We observed GI illness without a specified pathogen to dominate the ED visits attributable to waterborne pathogens, with only 1% of the burden attributed to specific pathogens. For hospitalisations and deaths, however, the burden attributed to specific pathogens was higher (36% and 60%, respectively), which suggests a higher likelihood of pathogen testing for more severe outcomes. The importance of unspecified pathogen GI illness has also been noted for foodborne illness [20]. Our estimates for hospitalisations and deaths for unspecified GI illness are in line with unspecified agent estimates reported in two studies [20, 21] that we scaled for waterborne transmission (see Table 3).

Two studies used administrative data to estimate the disease due to 13 pathogens that can be transmitted by water in the USA [16, 22]. There was overlap with our study for nine pathogens (*Campylobacter* spp., *Cryptosporidium* spp., *E. coli*, *Giardia* spp., *Legionella* spp., NTM, *Pseudomonas* spp., *Salmonella* spp. and *Shigella* spp.). In contrast to our study, the authors reported 100% of ED visits, hospitalisations and deaths that were linked with a pathogen (we applied a lower percentage, reflecting the portion attributable to water), counted any diagnosis type/cause of death (we used the range from only the principal diagnosis/underlying cause of death to any diagnosis type/cause of death) and did not estimate unspecified pathogen GI illness (we did). In terms of total waterborne illness, one study's hospitalisation rate was 3.2 times higher [22] and another study's death rate was 4.5 times higher [16] than the present study (Table 3). Corresponding estimates were in closer agreement for *Legionella* spp., for which nearly all the burden could be attributed to water. In contrast, the estimates were more divergent if other transmission routes were important (e.g. *Pseudomonas* spp.). The higher rates for NTM, for which most of the burden could be attributable to water, may represent true differences in incidence.

*Legionella* spp., NTM and *Pseudomonas* spp. were substantial contributors to waterborne hospitalisations and deaths. From Table 1 of Adam *et al*. [22] and Table 5 of Gargano *et al*. [16], 69% of hospitalisations and 91% of deaths were associated with these three pathogens. Our corresponding estimates were 82% of hospitalisations and 95% of deaths from all identified pathogens, and 29% of hospitalisations and 58% of deaths from the overall waterborne burden, indicating the large contribution of unspecified pathogen GI illness to the total burden in our analysis. Unlike enteric pathogens, *Legionella* spp., NTM and *Pseudomonas* spp. may not be adequately controlled by disinfection occurring at a treatment plant or other location within the municipal distribution system. These three pathogens are distinct from enteric pathogens with respect to their health effects and strategies for preventing disease. A QMRA study focused on potential activities using roof-harvested rainwater and indicated that drinking water, showering and garden hosing pose the highest risks for *Legionella* spp. and NTM infection [42]. They tend to inhabit biofilms in pipes and fixtures, such as showerheads and faucets, and in other non-potable water reservoirs which can then serve as sources of infections [11, 32, 43]. Establishing water system management programmes can help control these pathogens, through actions such as maintaining water temperatures outside the optimal growth range, altering plumbing to minimise water stagnation or low flow rates, and enhancing plumbing disinfection [44, 45].

In Ontario, data on 70 reportable diseases are collected from clinicians, laboratories and others, and disease cases are investigated by local public health units [46]. During case follow-up, information on whether the individual was hospitalised or died due to the disease is recorded. While reportable disease information reflects all transmission pathways, *Legionella* spp. transmission occurs predominantly via water. We note the estimated hospitalisation and death rates for *Legionella* spp. from this analysis were lower than corresponding reportable disease estimates (Table 3), though counts were small (e.g. 7 *vs.* 11 deaths). Previous investigators have compared administrative data and reportable disease data in Ontario. A study [47] found neither data source to be complete and both to underestimate the true incidence of pertussis, while another study [48] found that administrative data reasonably approximated reportable disease data for influenza hospitalisations. It is likely that the agreement between administrative and reportable disease data varies by disease, with both data sources underestimating the true burden of disease due to medical care not always being sought.

Another comparison of interest is the burden of disease transmitted by food *vs.* water. Drudge *et al*. [38] used a nearly identical approach and data sources to examine the burden of foodborne illness from 11 pathogens, food poisoning and unspecified GI illness in Ontario. Overall, the foodborne illness burden was larger than the waterborne burden by a factor of 4.2 for hospitalisations (Table 3). However, the food and water estimates were similar for deaths, which may be due to the substantial contribution of NTM to the waterborne totals.

As with other burden of disease studies, our study has several limitations. Our burden results are heavily dependent on the proportion of disease attributable to waterborne transmission (i.e. the attributable fraction). These inputs varied by pathogen and were associated with some degree of uncertainty. We attempted to account for this in our Monte Carlo simulation.

There was more information available for some pathogens than others to develop our attributable fraction estimates. In particular, the estimates for identified enteric pathogens were derived from an expert elicitation of 31 experts [28]. The mean water attributable fractions reported in other studies for *Campylobacter* spp. (range: 3–11%) and *Giardia* spp. (range: 41–42%) are in-line with our estimates, while our mean water attributable fraction for *Salmonella* spp. was higher than in other studies (range: 1–5%) [9, 49, 50]. The attributable fraction input we developed for unspecified pathogen GI illness is based on five Canadian estimates [8, 9, 35–37]. Given the lack of available estimates, we had to develop *de novo* attributable fraction estimates for *Legionella* spp., NTM and *Pseudomonas* spp. We based these attributable fraction estimates on available literature on environmental sources and discussion with clinical experts.

In terms of inputs for the burden analysis, administrative databases provided comprehensive counts for ED visits and hospitalisations. However, these data are collected by hospitals for financial and administrative management purposes, not specifically for tracking disease cases and causes, and so data may vary in accuracy across diseases or between medical coders [51]. For instance, previous studies have tried to account for the underestimation and misclassification of pathogen-specific disease. In a foodborne burden of disease analysis, Thomas *et al*. [21] applied under-ascertainment multipliers ranging from 1.3 to 16.8 to account for incompleteness in administrative data as well as underdiagnosis due to laboratory testing and test sensitivity. Using such multipliers in our analysis would likely lead to an increase in pathogen-specific burden, particularly for those pathogens that are not routinely tested for, as Thomas *et al*. [21] found. We chose not to use such multipliers because we required a consistent analytic approach across diverse outcomes that might result from exposure to environmental hazards (e.g. air pollution) considered as part of our mandate. While our results may underestimate specific pathogens and overestimate unspecified pathogens, they also demonstrate the burden unaccounted for through pathogen-focused surveillance systems.

Our estimates likely underestimate the overall amount of waterborne disease because our approach did not include the cases of disease where medical care was not sought or include an exhaustive list of illnesses transmitted by water, such as otitis media, infections due to *Vibrio* spp. or free-living amoebae. The persistence of pathogenic human viruses within free-living amoeba, in particular, has recently been identified as a public health concern [52].

Other metrics may illustrate different aspects of the burden of waterborne disease. Acute GI illness cases [8] will capture cases that may not result in healthcare system utilisation given their self-limited nature. Disability-adjusted life years combine mortality and morbidity into one metric, despite being difficult to interpret [23, 53, 54]. Cost estimates attempt to capture direct (e.g. medication) and sometimes indirect (e.g. lost days of work) costs associated with waterborne illness (e.g. [22, 55–57]). Similarly, other approaches to burden estimation, like a QMRA model or epidemiological study [7], may illustrate different aspects of waterborne disease. However, these approaches were not well suited to communicating to a broad lay audience. In our study, death and healthcare utilisation represent robust markers of moderate-to-severe disease and are widely understood by the public, the media and decision-makers. These metrics are also comparable across the range of environmental hazards (e.g. air pollution, temperature) in our larger environmental burden of disease project, so they were the best choice for our analysis. Although our estimates are for Ontario, we suspect they are not appreciably different from what would be found in neighbouring states and provinces and the methods we have used can be applied by others to their own jurisdiction's data.

Finally, our analysis examined population-wide health outcomes and presented plausible ranges of estimates for Ontario, which is useful for provincial-level ranking. It did not focus on sub-populations that may have unique exposure concerns. For example, many residents in Canadian Indigenous communities experience frequent drinking-water advisories, particularly where facilities rely on surface water as the source [58]. It should also be noted that the subset and ranking of pathogens may be different than for water, sanitation and hygiene concerns in other parts of the world, such as in low- and middle-income countries [53]. Temporal trends could be examined in future work since at least one study found lower drinking-water intake temperature was associated with a higher risk of GI illness hospitalisation in persons over the age of 65 [59].

Despite these limitations, this work provides the first estimates (to our knowledge) of waterborne disease that include GI illness from unspecified pathogens and pathogens transmitted through inhalation of water aerosols, and account for the proportion of disease transmitted by water. It highlights the importance of *Legionella* spp., NTM and *Pseudomonas* spp., for which current drinking-water treatment may not be adequate to reduce the risk to water users. This work also highlights the importance of GI illness without an identified pathogen. Pathogen-based surveillance systems will not pick up these cases, though syndrome-based surveillance (e.g. tracking based on a constellation of symptoms rather than laboratory-confirmed disease) may be able to. These findings may inform studies that failed to find associations between specific pathogens and outcomes of interest (e.g. a recent study did not find specific pathogens to be associated with recreational waterborne GI illness [60]).

## Conclusions

We estimated the burden of disease from waterborne pathogens in Ontario, Canada, including GI illness that was not traced back to a particular pathogen. We identified unspecified GI illness and the opportunistic pathogens *Legionella* spp., NTM and *Pseudomonas* spp. as key contributors to waterborne disease burden. Our study can inform decision-makers about the relative importance of enteric and non-enteric infections to the waterborne disease burden, and the need to address the latter with actions on infectious water aerosols. It also provides a template for other jurisdictions wishing to undertake a similar analysis.

## Supplementary material

For supplementary material accompanying this paper visit https://doi.org/10.1017/S0950268820000631. click here to view supplementary material
