# Supplementary material for: Estimates of healthcare utilisation and deaths from waterborne pathogen exposure in Ontario, Canada
Source: Epidemiol Infect. 2020 Mar 13;148:e70. doi: 10.1017/S0950268820000631 (PMC7118719; doi:10.1017/S0950268820000631)
Supplement: Supplementary file 1 [file S0950268820000631sup001.docx]

# Appendix A: Supplementary Input Data and Results

Table A1. Distribution and parameters for waterborne attributable fractions (%)

| Disease | Distribution | Parameters |
| --- | --- | --- |
| Adenovirus intestinal infection | Beta^a^ | Alpha1, alpha2, minimum, maximum:  2.29, 4.25, -1.02, 42.85 |
| *Campylobacter* spp. intestinal infection (campylobacteriosis) and associated GBS | Beta^a^ | Alpha1, alpha2, minimum, maximum:  2.63, 6.15, -0.97, 41.74 |
| *Cryptosporidium* spp. intestinal infection (cryptosporidiosis) | Beta^a^ | Alpha1, alpha2, minimum, maximum:  11.34, 20.05, -17.31, 114.68 |
| *Giardia* spp. intestinal infection (giardiasis) | Beta^a^ | Alpha1, alpha2, minimum, maximum:  10.48, 15.21, -2.73, 109.98 |
| *Legionella* spp. infection (legionellosis) | Uniform | Minimum, maximum:  95, 100 |
| NTM infection | Uniform | Minimum, maximum:  95, 100 |
| Norovirus intestinal infection | Beta^a^ | Alpha1, alpha2, minimum, maximum:  2.40, 7.78, -0.70, 43.89 |
| *Pseudomonas* spp. pneumonia and sepsis | Uniform | Minimum, maximum:  15, 35 |
| *Salmonella* spp. (nontyphoidal) infection (salmonellosis) | Beta^a^ | Alpha1, alpha2, minimum, maximum:  2.25, 6.93, -0.83, 55.21 |
| *Shigella* spp. intestinal infection (shigellosis) | Beta^a^ | Alpha1, alpha2, minimum, maximum:  2.33, 5.04, -1.12, 52.43 |
| *Toxoplasma gondii* infection (toxoplasmosis) | Lognormal^a^ | Mean, standard deviation, shift:  19.70, 7.48, -7.64 |
| VTEC intestinal infection and –associated HUS | Beta^a^ | Alpha1, alpha2, minimum, maximum:  3.42, 5.14, -2.79, 42.79 |
| Unspecified pathogen GI illness | Beta | Alpha1, alpha2, minimum, maximum:  1.93, 17.07, 0, 100 |

All distributions were truncated at 0% and 100%.

GBS, Guillain-Barré syndrome; GI, gastrointestinal; HUS, haemolytic-uraemic syndrome; NTM, nontuberculous mycobacteria; VTEC, verotoxin-producing *Escherichia coli*

^a^Developed by constraining the waterborne transmission route by the other major transmission routes in Butler et al. 2015.

Table A2a. Death counts in Ontario (corresponding to ICD-10 codes in Table 1) for the years 2012-2016

| Disease | Count (minimum/maximum)^a^ |  |  |  |  | Greatest % change from 2016^b^ |
| --- | --- | --- | --- | --- | --- | --- |
|  | 2012 | 2013 | 2014 | 2015 | 2016 |  |
| Adenovirus intestinal infection | –/– | –/– | –/– | –/– | –/– | –/– |
| *Campylobacter* spp. intestinal infection (campylobacteriosis) | –/– | –/– | –/– | –/– | –/– | –/– |
| *Campylobacter*-associated GBS | –/– | –/– | –/– | –/– | –/– | –/7 |
| *Cryptosporidium* spp. intestinal infection (cryptosporidiosis) | –/– | –/– | –/– | –/– | –/– | –/– |
| *Giardia* spp. intestinal infection (giardiasis) | –/– | –/– | –/– | –/– | –/– | –/– |
| *Legionella* spp. (legionellosis) | –/– | 9/20 | –/6 | 7/8 | –/8 | –/– |
| NTM infection | –/15 | 15/42 | 14/54 | 13/35 | 13/49 | –/69% |
| Norovirus intestinal infection | 11/17 | 6/9 | –/6 | –/7 | –/– | –/–% |
| *Pseudomonas* spp. pneumonia and sepsis | 6/41 | 13/39 | 9/36 | 10/41 | 7/47 | 86%/23% |
| *Salmonella* spp. (nontyphoidal) infection (salmonellosis) | –/– | –/– | –/– | –/6 | –/6 | –/– |
| *Shigella* spp. intestinal infection (shigellosis) | –/– | –/– | –/– | –/– | –/– | –/– |
| *Toxoplasma gondii* infection (toxoplasmosis) | –/– | –/– | –/– | –/– | –/– | –/– |
| VTEC intestinal infection | –/– | –/– | –/– | –/– | –/– | –/– |
| VTEC-associated HUS | –/– | –/– | –/– | –/– | –/– | –/– |
| Unspecified pathogen GI illness | 157/482 | 177/459 | 191/460 | 159/452 | 165/399 | 16%/21% |

GBS, Guillain-Barré syndrome; GI, gastrointestinal; HUS, haemolytic-uraemic syndrome; NTM, non-tuberculous mycobacteria; VTEC, verotoxin-producing *Escherichia coli*

^a^Counts with small cell sizes (<6) were suppressed; indicated by a dash.

^b^Absolute value.

Table A2b. Hospitalization counts in Ontario (corresponding to ICD-10-CA codes in Table 1) for the years 2012-2016

| Disease | Count (minimum/maximum)^a^ |  |  |  |  | Greatest % change from 2016^b^ |
| --- | --- | --- | --- | --- | --- | --- |
|  | 2012 | 2013 | 2014 | 2015 | 2016 |  |
| Adenovirus intestinal infection | 29/57 | 37/50 | 18/25 | 27/40 | 31/57 | 42%/56% |
| *Campylobacter* spp. intestinal infection (campylobacteriosis) | 177/235 | 171/216 | 174/217 | 143/189 | 140/176 | 26%/34% |
| *Campylobacter*-associated GBS | 56/86 | 48/80 | 64/100 | 66/99 | 64/91 | 25%/13% |
| *Cryptosporidium* spp. intestinal infection (cryptosporidiosis) | 6/14 | –/13 | 12/22 | 7/15 | –/11 | –/100% |
| *Giardia* spp. intestinal infection (giardiasis) | 6/27 | 11/32 | 10/24 | 9/25 | 11/25 | 45%/28% |
| *Legionella* spp. (legionellosis) | 102/145 | 135/205 | 76/100 | 73/96 | 81/120 | 67%/71% |
| NTM infection | 51/263 | 75/312 | 83/390 | 67/394 | 78/455 | 35%/42% |
| Norovirus intestinal infection | 51/157 | 32/119 | 64/179 | 54/162 | 44/109 | 45%/64% |
| *Pseudomonas* spp. pneumonia and sepsis | 350/1,047 | 340/1,021 | 358/1,082 | 362/1,150 | 423/1,293 | 20%/21% |
| *Salmonella* spp. (nontyphoidal) infection (salmonellosis) | 302/374 | 269/333 | 276/373 | 299/397 | 290/386 | 7%/14% |
| *Shigella* spp. intestinal infection (shigellosis) | 19/29 | 16/28 | 22/31 | 18/26 | 24/36 | 33%/28% |
| *Toxoplasma gondii* infection (toxoplasmosis) | –/19 | –/23 | –/23 | 7/32 | –/27 | –/30% |
| VTEC intestinal infection | 6/8 | 8/11 | –/7 | 12/12 | 13/16 | –/56% |
| VTEC-associated HUS | 18/56 | 31/67 | 24/57 | 25/55 | 24/59 | 30%/15% |
| Unspecified pathogen GI illness | 7,154/15,680 | 7,295/16,141 | 7,209/16,049 | 7,501/16,691 | 7,106/16,525 | 6%/5% |

GBS, Guillain-Barré syndrome; GI, gastrointestinal; HUS, haemolytic-uraemic syndrome; NA, not available; NTM, non-tuberculous mycobacteria; VTEC, verotoxin-producing *Escherichia coli*

^a^Counts with small cell sizes (<6) were suppressed; indicated by a dash.

^b^Absolute value.

Table A2c. Emergency department visit count (corresponding to ICD-10-CA codes in Table 1) in Ontario for the years 2012-2016

| Disease | Count (minimum/maximum)^a^ |  |  |  |  | Greatest % change from 2016^b^ |
| --- | --- | --- | --- | --- | --- | --- |
|  | 2012 | 2013 | 2014 | 2015 | 2016 |  |
| Adenovirus intestinal infection | –/– | 8/9 | 8/8 | 8/8 | –/– | –/– |
| *Campylobacter* spp. intestinal infection (campylobacteriosis) | 193/203 | 158/168 | 166/193 | 159/173 | 169/184 | 14%/10% |
| *Campylobacter*-associated GBS | 24/37 | 19/25 | 19/31 | 25/32 | 25/31 | 18%/20% |
| *Cryptosporidium* spp. intestinal infection (cryptosporidiosis) | 14/14 | 13/15 | 15/15 | 13/15 | 19/23 | 32%/39% |
| *Giardia* spp. intestinal infection (giardiasis) | 34/40 | 32/40 | 26/30 | 37/55 | 30/35 | 23%/57% |
| *Legionella* spp. (legionellosis) | –/6 | –/6 | –/– | –/– | –/7 | –/– |
| NTM infection | 18/37 | 25/52 | 19/48 | 15/39 | 24/55 | 38%/33% |
| Norovirus intestinal infection | 30/34 | 24/27 | 35/37 | 41/44 | 32/37 | 28%/27% |
| *Pseudomonas* spp. pneumonia and sepsis | 26/38 | 29/90 | 25/45 | 27/41 | 31/45 | 19%/100% |
| *Salmonella* spp. (nontyphoidal) infection (salmonellosis) | 207/227 | 155/189 | 225/290 | 200/241 | 208/252 | 25%/25% |
| *Shigella* spp. intestinal infection (shigellosis) | 21/21 | 12/12 | 14/19 | 14/17 | 31/34 | 61%/65% |
| *Toxoplasma gondii* infection (toxoplasmosis) | –/8 | –/7 | 6/8 | –/9 | 7/9 | –/22% |
| VTEC intestinal infection | –/– | –/– | –/– | –/– | –/– | –/– |
| VTEC-associated HUS | 11/16 | 7/14 | –/13 | 16/18 | 6/7 | –/163% |
| Unspecified pathogen GI illness | 82,730/94,890 | 85,283/98,034 | 86,270/99,473 | 90,586/104,622 | 88,358/103,074 | 6%/8% |

GBS, Guillain-Barré syndrome; GI, gastrointestinal; HUS, haemolytic-uraemic syndrome; NA, not available; NTM, non-tuberculous mycobacteria; VTEC, verotoxin-producing *Escherichia coli*

^a^Counts with small cell sizes (<6) were suppressed; indicated by a dash.

^b^Absolute value.

Table A3. Estimated attributable ED visits, hospitalizations, and deaths to identified waterborne pathogens and unspecified waterborne pathogens causing GI illness in Ontario in the year 2016

| Diseases^a^ | Attributable ED visits^a^ | Attributable hospitalizations^a^ | Attributable deaths^a^ |
| --- | --- | --- | --- |
|  | Mean (5^th^, 95^th^ )^b^ | Mean (5^th^, 95^th^)^b^ | Mean (5^th^, 95^th^)^b^ |
| Unspecified pathogen GI illness | 9,500 (1,700, 21,700) | 1,000 (200, 2,500) | 29 (5, 70) |
| NTM infection | 33 (24, 47) | 200 (90, 360) | 30 (14, 46) |
| *Pseudomonas* spp. pneumonia and sepsis | <10 | 180 (90, 310) | 7 (2, 13) |
| *Legionella* spp. infection | <10 | 92 (80, 109) | 5 (2, 8) |
| *Sub-total for* opportunistic pathogens^c^ | 47 (36, 61) | 470 (310, 660) | 42 (25, 60) |
| *Salmonella* spp. (nontyphoidal) infection | 29 (6, 60) | 42 (8, 87) | <1 |
| *Campylobacter* spp.-intestinal infection and -associated GBS | 24 (6, 47) | 27 (6, 54) | 1 (0, 2) |
| *Giardia* spp. intestinal infection | 14 (8, 20) | <10 | <1 |
| Sum of pathogens below presentation threshold^d^ | 17 (11, 24) | 27 (17, 40) | 1 (0, 1) |
| *Sub-total all identified pathogens* | *130 (96, 171)* | *570 (400, 770)* | *44 (27, 61)* |
| *Sub-total all enteric pathogens^e^* | *84 (51, 122)* | *102 (59, 156)* | *2 (1, 3)* |
| *Total* | *9,600 (1,900, 21,900)* | *1,600 (700, 3,100)* | *73 (41, 117)* |

ED, emergency department; GBS, Guillain-Barré syndrome; GI, gastrointestinal; NTM, nontuberculous mycobacteria.

^a^Results reported when the attributable counts were at least 10 ED visits, 10 hospitalizations, or 1 death. The sum of the pathogens that did not meet the presentation threshold is shown as a separate row. Results may not add up due to rounding.

^b^The simulation is summarized by the mean and 5^th^ and 95^th^ percentiles of the 10,000 iterations.

^c^This is the sub-total for *Legionella* spp. infection, NTM infection, and *Pseudomonas* spp. pneumonia and sepsis from the rows above.

^d^The sum for the six pathogens that were below the presentation threshold: adenovirus, *Cryptosporidium* spp., norovirus, *Shigella* spp., *Toxoplasma gondii*, and verotoxin-producing *Escherichia coli*.

^e^ This is a sum of all enteric pathogens: adenovirus, Campylobacter spp., Cryptosporidium spp., Giardia spp., norovirus, Salmonella spp., Shigella spp., Toxoplasma gondii, and verotoxin-producing *Escherichia* coli

Note: we estimated attributable physician office visits for unspecified GI illness as 38,000 (5^th^ and 95^th^ percentiles: 7,000, 87,900)
